# What is the effect of interrupting prolonged sitting with frequent bouts of physical activity or standing on first or recurrent stroke risk factors? A scoping review

**DOI:** 10.1371/journal.pone.0217981

**Published:** 2019-06-13

**Authors:** Paul Mackie, Ishanka Weerasekara, Gary Crowfoot, Heidi Janssen, Elizabeth Holliday, David Dunstan, Coralie English

**Affiliations:** 1 School of Health Sciences and Priority Research Centre for Stroke and Brain Injury, University of Newcastle, Newcastle, Australia; 2 Centre for Research Excellence in Stroke Recovery and Rehabilitation, Florey Institute of Neuroscience, Melbourne, Australia; 3 Hunter Stroke Service, Hunter New England Local Health District, Newcastle, Australia; 4 School of Medicine and Public Health University of Newcastle, Newcastle, Australia; 5 Baker Heart and Diabetes Institute, Melbourne, Australia; 6 Mary MacKillop Institute for Health Research, Australian Catholic University, Melbourne, Australia; University of Toronto, CANADA

## Abstract

The objective of this review was to ascertain the scope of the available literature on the effects of interrupting prolonged sitting time with frequent bouts of physical activity or standing on stroke and recurrent stroke risk factors. Databases Medline, Embase, AMED, CINAHL and Cochrane library were comprehensively searched from inception until 21^st^ February 2018. Experimental trials which interrupted sitting time with frequent bouts of physical activity or standing in adults (≥ 18 years) were included. Comparison to a bout of prolonged sitting and a measure of at least one first or recurrent stroke risk factor was required to be included. Overall, 30 trials (35 articles) were identified to meet the inclusion criteria. Fifteen trials were completed in participants at an increased risk of having a first stroke and one trial in participants at risk of a recurrent stroke. Outcomes of hypertension and dysglycemia were found to be more favourable following predominately light- to moderate-intensity bouts of physical activity or standing compared to sitting in the majority of trials in participants at risk of having a first stroke. In the one trial of stroke survivors, only outcomes of hypertension were significantly improved. These findings are of significant importance taking into consideration hypertension is the leading risk factor for first and recurrent stroke. However, trials primarily focused on measuring outcomes of dysglycemia and without assessing a dose-response effect. Additional research is required on the dose-response effect of interrupting sitting with frequent bouts of physical activity or standing on first and recurrent stroke risk factors, in those high risk population groups.

## Introduction

Engaging in high levels of sitting is associated with detrimental risks of all-cause mortality, cardiovascular disease and diabetes [1–3]. Spending > 8 hours/day in sitting and engaging in < 2.5 metabolic equivalents (MET—defined by Jette et al. [4] as “the amount of oxygen consumed while sitting at rest and is equal to 3.5 ml O_2_/kg/min”) hours/week of physical activity accounts for a 59% increase in all-cause mortality relative to individuals who sit < 4 hours/day and engage in > 35.5 MET hours/week [5].

Stroke survivors, a population at high risk of having recurrent strokes, spend a large proportion of their day sitting [6, 7]. Pooled data from the National Health and Nutrition Survey (NHANES) found that American stroke survivors spend 8.5% (weighted prevalence) more time sitting than those from non-stroke populations [7]. Stroke survivors spend on average 22% more time sitting than healthy age-matched controls [6]. The high amount of time spent sitting likely augments the already compromised health and risk of stroke survivors.

Interventions that target specific modifiable risk factors associated with first and recurrent stroke risk could aid in improving the health of stroke survivors and reducing the risk of first and recurrent strokes. In a recent case-control study (n = 26,919) [8], 91% of the population attributable risk (PAR) for first stroke was associated with 10 modifiable factors (PAR: hypertension 48%, physical inactivity 36%, lipids 27%, poor diet 23%, waist to hip ratio 19%, psychosocial 17%, cardiac 9%, alcohol 6%, diabetes 4%). Risk of recurrent stroke was associated with six modifiable factors (hypertension, smoking, high cholesterol, glycated haemoglobin (HbA1c), low physical activity and weight management) [9]. Interventions which incorporate physical activity have the potential to reduce these first and recurrent stroke risk factors [10, 11]. However, only 18% of stroke survivors meet the recommended guidelines for physical activity (150 minutes/week of moderate-intensity physical activity) [7]. With such context, combined with the known susceptibility of stroke survivors to sit for large periods of the day[6], new paradigms such as breaking up prolonged sitting time may be a promising strategy to reduce the risk of recurrent strokes.

Experimental studies have shown that frequently breaking up sitting time with physical activity or standing bouts has beneficial effects on cardio-metabolic health in non-stroke populations [12–14]. Frequent bouts of light- or moderate-intensity walking, simple resistance activities or cycling, have been shown to attenuate the exaggerated postprandial glucose and insulin, and blood pressure response to prolonged sitting [14, 15], in those with type 2 diabetes [16, 17], postmenopausal women [18], overweight/obese [12, 15] and healthy [19]. In the first ever study in-stroke survivors, 3-minute bouts of light-intensity exercises while standing (STAND-EX), performed every 30 minutes, resulted in significant reductions in systolic blood pressure (3.5 mmHg) when compared to 8 hours of prolonged sitting [20]. However, in order to inform research development and subsequently promote effective clinical interventions, evidence is required regarding the effect of breaking up sitting time on first or recurrent stroke risk factors.

Reviews have previously investigated the benefits of interrupting sitting time with frequent bouts of physical activity or standing on markers associated with cardio-metabolic health, obesity and all-cause mortality [21–24]. However, they did not focus on outcome measures associated with first and recurrent stroke risk or identify population groups primarily targeted. Therefore, the aim of this study was to review the evidence for the effect of interrupting prolonged sitting with frequent bouts of physical activity or standing on first or recurrent stroke risk factors. Specifically, our research questions were:

- What are the characteristics of population groups assessed?
- What are the characteristics of the physical activity or standing bouts used (type, duration, frequency, intensity)?
- What first or recurrent stroke risk factors have been measured?
- What are the effects of frequent bouts of physical activity or standing on first or recurrent stroke risk factors?

## Methods

The methodological framework by Arksey and O’Malley [25] and further recommendations from Levac et al. [26] were utilised in this scoping review. The stages underpinning the review were: (I) identifying the research question, (II) identifying relevant studies, (III) study selection, (IV) charting the data, and (V) collating, summarising, and reporting the results. The quality of studies was not assessed in this review as per recommendations by Arksey and O’Malley [25].

### Identification of the research question

The four stage PICO format (Population, Intervention, Comparison, and Outcome) was used to design and define the research question. The section below clarifies each aspect of the research question.

#### Population

Trials had to be conducted in adult (aged ≥ 18 years) males and females. Adults included stroke and non-stroke population groups.

#### Intervention and comparison

The intervention(s) in each trial had to involve frequent (≥ 2) bouts of physical activity or standing, and include a comparison of uninterrupted, prolonged sitting. Interventions had to be supervised to ensure protocol adherence. Supervision was defined as whereby participants were observed, monitored or supervised throughout conditions, or where interventions were conducted within a research facility (e.g. laboratory setting). There were no restrictions placed on the type (e.g. walking, standing, cycling), duration, frequency or intensity of physical activity bouts.

#### Outcome

Trials were required to include a measure of at least one risk factor associated with first or recurrent stroke risk. First and recurrent stroke risk factors identified from the INTERSTROKE case control trial [8], the Global Burden of Disease Study (2013) [27] and the Stenting and Aggressive Medical Management for Preventing Recurrent Stroke in Intracranial Stenosis [9] are reported in Table 1.

**Table 1:** Risk factors for first and recurrent stroke.

**First stroke**	
***Risk factors***	***Outcome measures***
Hypertension	High systolic blood pressure [8, 27]
	High diastolic blood pressure
	High mean arterial pressure [28]
Dysglycemia	High fasting plasma glucose [27]
	Abnormal post-prandial glucose
	Impaired glucose tolerance [29]
	High HbA1c or self-reported diabetes [8]
Anthropometric risk	High BMI [27]
	Waist-to-hip ratio [8]
Hypercholesterolaemia	High total cholesterol [27]
	ApoB/ApoA 1 ratio [8]
Behavioural risks [8, 27]	Poor diet
	Smoking
	Low physical activity
	High alcohol intake
Psychosocial risks [8]	Psychosocial stress
	Depression
Cardiac risks [8]	Atrial fibrillation
	Myocardial infarction
**Recurrent stroke** [9]	
Hypertension	High systolic blood pressure
Dysglycemia	High HbA1c
Anthropometric risk	Weight management High BMI Weight loss
Hypercholesterolaemia	High LDL-C
	High HDL-C
Behavioural risks	Smoking
	Low physical activity

ApoB/ApoA 1 ratio, apolipoproteinB/apolipoproteinA1 ratio; BMI, body mass index; HbA1c, glycated haemoglobin; HDL-C, high density lipoprotein cholesterol; LDL-C, low density lipoprotein cholesterol

### Identifying relevant studies for selection

#### Search strategy

The search strategy developed was guided by a Hunter New England Health librarian and revised by the research team. It was developed in Medline (Ovid) and adapted to other relevant databases including Embase (Ovid), Allied and Complementary Medicine (AMED; Ovid), Cumulative Index to Nursing and Allied Health Literature (CINAHL; EBSCOhost) and the Cochran library (Wiley). Databases were searched comprehensively from the date of inception to 14^th^ July 2017. A final search was completed on the 21^st^ February 2018.

Relevant trial registries were also searched for unpublished trials and to assist in identifying the trials which had been published across several articles.

Search terms included Medical Subject Headings (MeSH) and keywords related to, but not limited to, “sedentary behaviour” (e.g. sitting, sedentary lifestyle, uninterrupted) and “interventions” (e.g. bouts, walking, standing). The full Medline search strategy is included in S1 Appendix. Restrictions on searches were limited to *English language* and *humans*. The PRISMA checklist made relevant to this scoping review is included in S2 Appendix.

#### Eligibility criteria

To be included, studies had to meet the following criteria: (I) include a supervised intervention of interrupting sitting time with frequent bouts of physical activity or standing (experimental studies), (II) involve human adult participants (age ≥ 18 years), (III) be written in English, and (IV) include at least one outcome measure related to a first or recurrent stroke risk factor. Exclusion criteria included: (I) non-experimental (e.g. observational, case-control, cross-sectional, longitudinal) studies investigating associations of sedentary behaviour and activity bouts (without implementation of an intervention). Relevant reviews (systematic and met-analysis) identified were excluded, but reference lists were hand-searched to identify additional eligible articles.

Title and abstract (PM and GC), and full text screening (PM and IW) were completed separately, with each article independently screened by the principal investigator (PM) and other members of the research team (IW and GC). Discrepancies during screening and reviewing were resolved by a third member of the research team (CE).

### Charting the data for extraction

Data extraction was independently completed by two reviewers (PM and IW). The data extraction spreadsheet was designed to capture all relevant details required to answer the research questions and included: author, year published, sample size, population characteristics (e.g. age, comorbidities, anthropometrics), outcome measures associated with first and recurrent stroke risk factors (see Table 1), assessment times of outcomes (e.g. frequency of measures and on which assessment day), study length (e.g. number of days), physical activity bout type (e.g. walking, standing), frequency (how often bouts were completed), duration and intensity, and study setting (e.g. laboratory, workplace). The spreadsheet was refined via an iterative process in collaboration with the two reviewers.

### Collating, summarising and reporting the results

Descriptive analysis of the data extracted was undertaken to describe the nature of the studies and to answer the research question.

## Results

**Fig 1:**
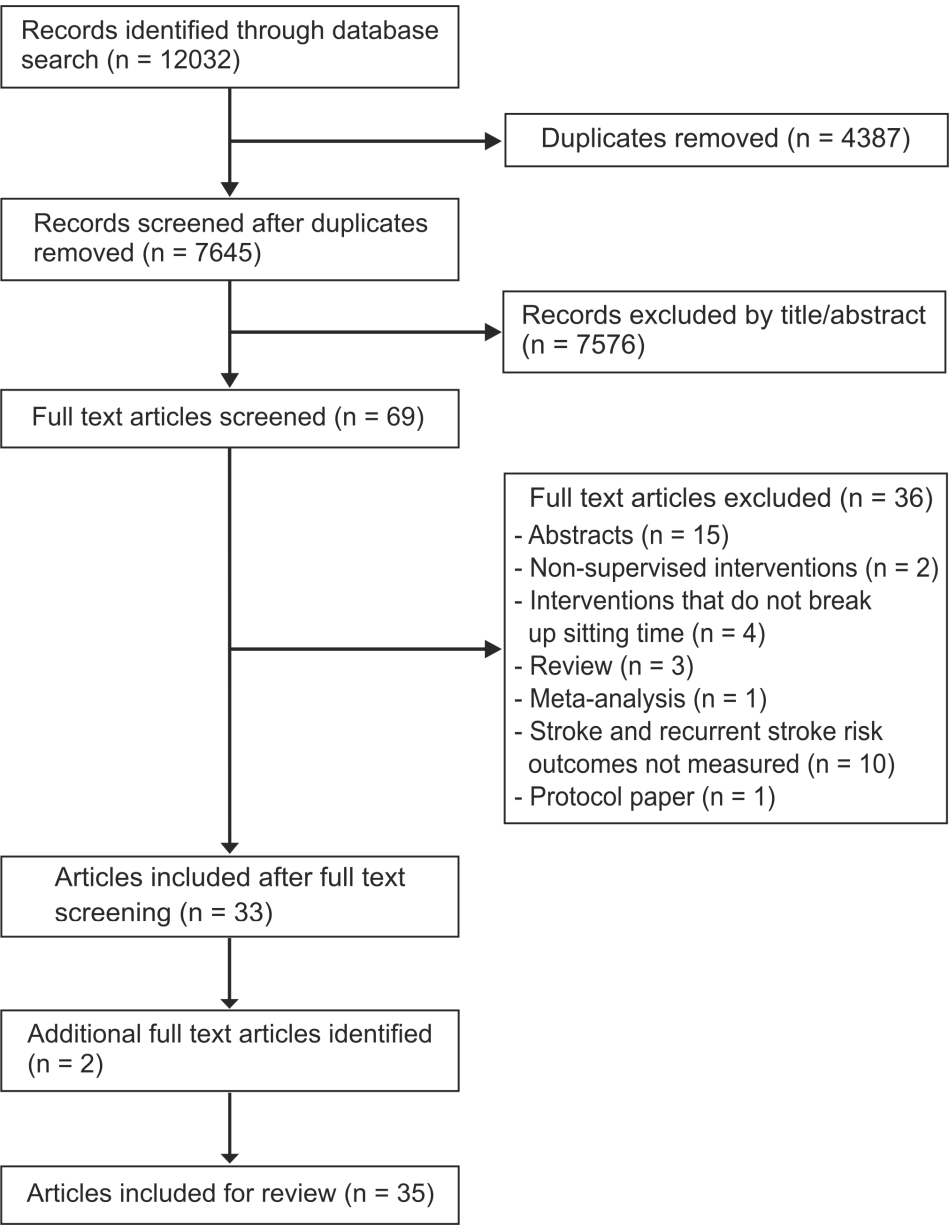
Flow chart of database search.

A total of 29 trials (33 articles) were identified in our search as meeting all inclusion criteria (Fig 1). One (two articles [20, 30]) additional trial was included on the 13^th^ June 2018. Therefore, a total of 30 trials (35 articles) were included in this review, of which 53% were not registered with a trial registry.

All trials used a randomised crossover design, expect for one which used a balanced crossover design [31]. In 20 trials, interventions occurred in a laboratory or research facility where participants were supervised during each condition [12, 14, 16, 18, 20, 30, 32–50]. One trial was completed under supervised conditions in an office setting [51] and nine trials were conducted in a laboratory or research facility [19, 31, 52–58] where participants where not reported to be observed during conditions.

### Characteristics of population groups

Participants of included studies were categorised into five distinct groups: (I) healthy adults, (II) overweight/obese adults, (III) individuals with type 2 diabetes, (IV) postmenopausal women, and (V) people with stroke.

Of the 30 trials included, 14 trials [19, 31, 32, 39–41, 43, 47, 48, 51–53, 56, 57] specifically recruited healthy adults. The characteristics of the included participants is summarised in Table 2. Notably, eight trials [19, 31, 32, 39, 43, 47, 53, 57] included adults of normal weight (Body mass index: BMI < 25 kg.m^-2^) and three trials [41, 48, 51] included overweight participants (BMI ≥ 25 kg.m^-2^ and < 29.9 kg.m^-2^). The age groups ranged from 21 years to 52 ± 5 years, with the primary focus (79%) being in young adults aged ≥ 18 years and ≤ 35 years (11 trials [19, 31, 32, 40, 41, 43, 47, 52, 53, 56, 57]).

**Table 2:** Summary of included trials.

Authors (Year) Trial ID	Design	Population	Age (years)	N	Protocol	Outcomes	Results
**Healthy**							
Homer et al. (2017) *ANZCTR12614000624684*	Randomised crossover trial	Healthy, normal weight adults 25 females, 11 males BMI = 23.7 (4.0)	25 (19–34)*	36	4 conditions, each conducted over 2 days: 1. Uninterrupted sitting a. Duration: Day 1; 7 hours, Day 2; 5 hours 2. Physical activity bouts (Day 1) a. Type: WALK b. Duration: 30 minutes c. Frequency: Single bout d. Intensity: 60% VO2_max_ 3. Physical activity bouts (Day 1 and Day 2) a. Type: WALK b. Duration: 2 minutes c. Frequency: every 30 minutes d. Intensity: 60% VO2_max_ 4. Physical activity bouts (Day 1 and 2) a. Type: WALK b. Duration: intermittent walking; 2 minutes, single bout; 30 minutes c. Frequency: intermittent walking; every 30 minutes, single bout; end of day d. Intensity: 60% VO2_max_	II. Dysglycemia	**Fasting glucose:** No significant effects of conditions on Day 2 fasting glucose concentrations (*p* = 0.20) **Postprandial glucose:** No significant effect of conditions on postprandial glucose concentrations (*p* = 0.29)
Peddie et al. (2013) *ACTRN12610000953033*	Randomised crossover trial	Healthy, normal weight adults working in a predominately sedentary occupation 42 females, 28 males BMI = 23.6 (4.0)	26 (5)	70	3 conditions 1. Uninterrupted sitting a. Duration: 9 hours 2. Physical activity bouts a. Type: WALK b. Duration: 30 minutes c. Frequency: single bout d. Intensity: 60% VO2_max_ 3. Physical activity bouts a. Type: WALK b. Duration: 1 minute 40 seconds c. Frequency: every 30 minutes d. Intensity: 60% VO2_max_	II. Dysglycemia	**Postprandial glucose:** A significant condition effect was found for glucose iAUC (*p* < 0.01) Regular activity bouts significantly reduced glucose iAUC (*p* < 0.01) compared to uninterrupted sitting and a single bout of physical activity
Benatti et al. (2017) *NCT02215603*	Randomised crossover trial	Healthy, physically inactive males BMI = 24.9 (4.3)	30 (9)	14	4 conditions 1. Uninterrupted sitting a. Duration: 9 hours 2. Physical activity bouts a. Type: STAND b. Duration: 15 minutes c. Frequency: every 30 minutes a. Intensity: not specified 3. Physical activity bouts a. Type: WALK b. Duration: 30 minutes c. Frequency: single bout d. Intensity: 50% - 55% VO2_max_ (moderate) 4. Physical activity bouts a. Type: WALK + STAND b. Duration: WALK; 30 minutes, STAND; 15 minutes c. Frequency: WALK; single bout, STAND; every 30 minutes d. Intensity: WALK; 50% - 55% VO2max (moderate)	Dysglycemia IV. Hypercholesterolemia	**Postprandial glucose:** Glucose iAUC (12 hour) was lower in STAND versus uninterrupted sitting (*p* = 0.04) **Total cholesterol** No significant differences **HDL cholesterol** No significant differences **LDL cholesterol:** No significant differences
McCarthy et al. (2017) *NCT02493309*	Randomised crossover trial	Healthy, non-obese adults working in a predominately sedentary occupation 18 females, 16 males BMI = 24.5 (3)	40 (9)	34	2 conditions 1. Uninterrupted sitting a. Duration: 7.5 hours 2. Physical activity bouts a. Type: WALK b. Duration: 5 minutes c. Frequency: every 30 minutes d. Intensity: 3 km.h^-1^ (light)	II. Dysglycemia	**Postprandial glucose:** A significant effect of condition was seen for glucose iAUC (*p* = 0.02) with walking bouts revealing a 35% reduction in iAUC compared to uninterrupted sitting
Brocklebank et al. (2017) *ISRCTN48132950*	Randomised crossover trial	Healthy office workers in a predominately sedentary occupation 9 females, 8 males BMI = 28.0 (4.5)	52 (5)	17	3 conditions 1. Uninterrupted sitting a. Duration: 5 hours 2. Physical activity bouts a. Type: STAND b. Duration: 2 minutes c. Frequency: every 20 minutes d. Intensity: Standing as still as possible 3. Physical activity bouts a. Type: WALK b. Duration: 2 minutes c. Frequency: every 20 minutes d. Intensity: RPE 9 (light)	II. Dysglycemia	**Postprandial glucose:** Walking bouts significantly reduced 5-hour iAUC by 55.5% lower compared to uninterrupted sitting (*p* = 0.02)
Miyashita et al. (2013) *No trial registry number*	Randomised crossover trial	Healthy, normo-lipidaemic men BMI = 22.5 (1.5)	27 (2)	15	3 conditions, each conducted over 2 days (Day 1; conditions, Day 2; uninterrupted sitting) 1. Uninterrupted sitting a. Duration: Day 1; 7.5 hours, Day 2; 6 hours 2. Physical activity bouts a. Type: STAND b. Duration: 45 minutes c. Frequency: every hour d. Intensity: not specified 3. Physical activity bouts a. Type: WALK b. Duration: 30 minutes c. Frequency: single bout d. Intensity:62 ± 3% age-predicted HR_max_	II. Dysglycemia	**Fasting glucose:** No significant differences **Postprandial glucose:** A significant main effect of condition was seen (*p* = 0.01). Postprandial glucose was significantly reduced after a single walking bout compared to uninterrupted sitting (*p* = 0.01)
Altenburg et al. (2013) *No trial registry number*	Randomised crossover trial	Healthy young adults 6 females, 5 males BMI = 23.2 (20.1–26.1)*	21 (20–23)*	11	2 conditions 1. Uninterrupted sitting a. Duration: 8 hours 2. Physical activity bouts a. Type: CYCLE b. Duration: 8 minutes c. Frequency: every hour d. Intensity: 40–60% HRR (moderate)	II. Dysglycemia IV. Hypercholesterolaemia	**Postprandial glucose:** No significant differences **Total cholesterol:** No significant differences **HDL cholesterol:** No significant differences **LDL cholesterol:** No significant differences
Carter & Gladwell. (2017) *No trial registry number*	Counter-balanced randomised trial	Healthy adults 4 females, 6 males BMI—not reported	27 (8)	10	2 conditions 1. Uninterrupted sitting a. Duration: 1 hour 26 minutes 2. Physical activity bouts a. Type: Calisthenics (squats, arm circles, calf raises, knees to elbow and lunges) b. Duration: 2 minutes c. Frequency: every 20 min d. Intensity: not specified	I. Hypertension	**Mean atrial pressure:** No significant differences
Bailey et al. (2016) *No trial registry number*	Randomised crossover trial	Healthy, inactive, sedentary adults 7 females, 6 males BMI—not reported	27 (9)	13	3 conditions 1. Uninterrupted sitting a. Duration: 5 hours 2. Physical activity bouts a. Type: WALK b. Duration: 2 minutes c. Frequency: every 20 minutes d. Intensity: 3.2 km.h^-1^ (light) 3. Physical activity bouts a. Type: WALK b. Duration: 2 minutes c. Frequency: every 20 minutes d. Intensity: between 5.8–7.9 km.h^-1^ (moderate)	II. Dysglycemia	**Postprandial glucose:** A significant effect of condition was seen (*p* < 0.01) with iAUC reduced during SIT + MA compared to SIT + LA (*p* < 0.01), but not compared to uninterrupted sitting (*p* = 0.06)
Bailey & Locke. (2015) *No trial registry number*	Randomised crossover trial	Healthy, non-obese adults 3 females, 7 males BMI = 26.5 (4.3)	24 (3)	10	3 conditions 1. Uninterrupted sitting a. Duration: 5 hours 2. Physical activity bouts a. Type: Stand b. Duration: 2 minutes c. Frequency: every 20 minutes d. Intensity: standing as still as possible 3. Physical activity bouts a. Type: WALK b. Duration: 2 minutes c. Frequency: every 20 minutes d. Intensity: 3.2 km.h^-1^ (light)	I. Hypertension II. Dysglycemia IV. Hypercholeserolaemia	**Systolic blood pressure:** No significant differences **Diastolic blood pressure:** No significant differences **Postprandial glucose:** Glucose AUC was significantly different between conditions (*p* <0.01) with walking bouts significantly reducing AUC (*p* < 0.01) by 16.7% and 15.9% compared to standing bouts and uninterrupted sitting, respectively **Total cholesterol:** No significant differences **HDL cholesterol:** No significant differences
Engeroff et al. (2017) *No trial registry number*	Balanced crossover trial	Healthy young premenopausal women BMI = 21.5 (2)	26 (3)	18	3 conditions 1. Uninterrupted sitting a. Duration: 4 hours 2. Physical activity bouts a. Type: CYCLE b. Duration: 6 minutes c. Frequency: every 40 minutes d. Intensity: 70% VO2_max_ 3. Physical activity bouts a. Type: CYCLE b. Duration: 30 minutes c. Frequency: single bout d. Intensity: 70% VO2_max_	IV. Hypercholesterolaemia	**Total cholesterol:** Significant trial x time interaction (*p* = 0.04). Change in BREAK condition significantly differed to changes in PRE condition (*p* = 0.01) **HDL cholesterol:** Significant trial x time interaction (*p* = 0.01). Change in BREAK condition significantly different to change in PRE (*p* = 0.01) and uninterrupted sitting (*p* = 0.03) **LDL cholesterol:** No significant differences
Hansen et al. (2016) *No trial registry number*	Randomised crossover trial	Healthy young, normal weight and recreationally active adults 8 females, 6 males BMI = 23 (21.6–24.4)*	22 (20–23)*	14	2 conditions 1. Uninterrupted sitting a. Duration: 2.5 hours 2. Physical activity bouts a. Type: WALK b. Duration: 2 minutes c. Frequency: every 20 minutes d. Intensity: 4.1 ± 0.3 kmh^−1^ (light)	II. Dysglycemia	**Postprandial glucose:** No significant differences
Kim et al. (2014) *No trial registry number*	Randomised crossover trial	Healthy young recreationally active males BMI—not reported	24 (4)	9	3 conditions, each conducted over 4 days (Day 1 and Day 2; stabilisation phase, Day 3; activity bout conditions, Day 4; High-fat tolerance test): 1. Uninterrupted sitting a. Duration: Day 3; 9 hours, Day 4; 7 hours 2. Physical activity bouts a. Type: RUN b. Duration: 60 minutes c. Frequency: single bout d. Intensity: 65% VO2_max_ 3. Physical activity bouts a. Type: WALK b. Duration: 30–60 minutes (average 17.8 ± 4 min) c. Frequency: every hour d. Intensity: 25% VO2_max_ (light)	II. Dysglycemia	**Fasting glucose:** No significant differences **Postprandial glucose:** Significant effect of treatment (*p* < 0.05). Plasma glucose was significantly reduced following LOW (*p* = 0.02) and MOD (*p* = 0.01) compared to uninterrupted sitting. MOD significantly lower compared to LOW (*p* = 0.03)
Pulsford et al. (2017) *No trial registry number*	Randomised crossover trial	Healthy, inactive, weight stable males BMI = 26.1 (4.1)	40 (12)	25	3 conditions 1. Uninterrupted sitting a. Duration: 7 hours 2. Physical activity bouts a. Type: STAND b. Duration: 2 minutes c. Frequency: every 20 minutes d. Intensity: standing still 3. Physical activity bouts a. Type: WALK b. Duration: 2 minutes c. Frequency: every 20 minutes d. Intensity: 2 mph (light)	II. Dysglycemia	**Postprandial glucose:** Significant condition effect (AUC; *p* < 0.01). AUC lower following SIT-WALK compared to uninterrupted sitting (*p* < 0.01) and SIT- STAND (*p* = 0.04)
**Overweight/obese**							
Dunstan et al. (2012) *ACTRN12609000656235* Latouche et al. (2013) Larsen et al. (2014)	Randomized crossover trial	Overweight/obese adults 8 females, 11 males BMI = 31.2 (4.1)	54 (5)	19	3 conditions 1. Uninterrupted sitting a. Duration: 5 hours 2. Physical activity bouts a. Type: WALK b. Duration: 2 minutes c. Frequency: every 20 minutes d. Intensity: 3.2 km.h^-1^ (light) 3. Physical activity bouts a. Type: WALK b. Duration: 2 minutes c. Frequency: every 20 minutes d. Intensity: 5.8–6.4 km.h^-1^ (moderate)	I. Dysglycemia	**Postprandial glucose:** Glucose iAUC (5-hours) was significantly reduced following light-intensity (*p* < 0.01) and moderate-intensity (*p* < 0.01) walking in comparison to uninterrupted sitting
		Subgroup 1 female, 7 males BMI = 30.9 (2.9)	55 (6)	8		II. Dysglycemia	**Postprandial glucose:** Glucose iAUC was significantly reduced after light-intensity (*p* < 0.01) and moderate-intensity (*p* = 0.02) walking compared to uninterrupted sitting
		Subgroup 8 females, 11 males BMI = 31.2 (0.9 SEM)	54 (1 SEM)	19		III. Hypertension	**Systolic blood pressure:** SBP was significantly reduced with light-intensity (*p* < 0.01) and moderate-intensity (*p* = 0.02) walking in comparison to uninterrupted sitting. In pre-hypertensive and hypertensive individuals, SBP was significantly reduced compared to uninterrupted sitting (*p* = 0.01) following light-intensity walking **Diastolic blood pressure:** DBP was significantly reduced with light-intensity (*p* = 0.01) and moderate-intensity (*p* = 0.03) walking compared to uninterrupted sitting. In pre-hypertensive and hypertensive individuals, DBP was significantly reduced compared to uninterrupted sitting (*p* < 0.01) following light-intensity walking. Light-intensity walking was no longer significant when those treated with antihypertensive therapy were removed **Mean arterial pressure:** No significant differences
Wennberg et al. (2016) *ACTRN12613000137796*	Randomised crossover trial	Overweight/obese adults 9 females, 10 males BMI = 31.5 (4.7)	60 (8)	19	2 conditions 1. Uninterrupted sitting a. Duration: 7 hour 2. Physical activity bouts a. Type: WALK b. Duration: 3 minutes c. Frequency: every 30 minutes d. Intensity: 3.2 km.h-1, mean RPE = 9.1 ± 2 (light)	I. Hypertension II. Dysglycemia	**Systolic blood pressure:** No significant differences **Diastolic blood pressure:** No significant differences **Postprandial glucose:** No significant difference
Thorp et al. (2014) *No trial registry number*	Randomised crossover trial	Overweight/obese sedentary office workers 6 females, 17 males BMI = 29.6 (4.1)	48 (8)	23	2 conditions each conducted over 5 days (assessments on Day 1 and Day 5) 1. Uninterrupted sitting a. Duration- 8 hours 2. Physical activity bouts a. Type: STAND b. Duration: 30 minutes c. Frequency: every 30 minutes d. Intensity: not specified	I. Dysglycemia III. Anthropometric	**Fasting glucose:** No significant differences **Postprandial glucose:** A significant difference between conditions was reported (*p* = 0.01) with adjusted iAUC (4 hour) lower following the standing bouts compared to uninterrupted sitting **Weight:** No significant differences
Larsen et al. (2015) *ACTRN12610000657022*	Randomised crossover trial	Overweight/obese, sedentary adults 8 females, 11 males BMI = 32.7 (1 SEM)	57 (2 SEM)	19	2 conditions 1. Uninterrupted sitting a. Duration: 7 hours 2. Physical activity bouts a. Type: WALK b. Duration: 2 minutes c. Frequency: every 20 minutes d. Intensity: 3.2 km.h^-1^, RPE = 6–11 (light)	II. Dysglycemia	**Fasting glucose:** No significant differences **Postprandial glucose:** WALK bouts significantly reduced glucose iAUC (*p* < 0.01) and tAUC (*p* = 0.01) on Day 1 and Day 3 compared to uninterrupted sitting
Zeigler et al. (2016) *No trial registry number*	Randomised crossover study	Overweight/obese and physically inactive (pre-hypertensive or with impaired fasting glucose) 7 females, 2 males BMI = 28.7 (2.7)	30 (15)	9	4 conditions 1. Uninterrupted sitting a. Duration: 8 hours 2. Physical activity bouts a. Types: STAND b. Duration:10–30 minutes c. Frequency: approximately every hour d. Intensity: not specified 3. Physical activity bouts a. Type: WALK b. Duration: 10–30 minutes c. Frequency: every hour d. Intensity: 1 mph 4. Physical activity bouts a. Type: CYCLE b. Duration: 10–30 minutes c. Frequency: every hour d. Intensity: 20W	I. Hypertension	**Systolic blood pressure:** STAND, CYCLE and WALK significantly reduced SBP compared to uninterrupted siting (all *p* < 0.01). CYCLE was significantly lower compared to WALK (*p* < 0.01) and STAND (*p* = 0.04) **Diastolic blood pressure:** CYCLE significantly reduced DBP compared to uninterrupted sitting (*p* < 0.01)
Bhammer et al. (2017) *No trial registry number*	Randomised crossover trial	Overweight/obese and physically inactive adults 5 females, 5 males BMI = 30.3 (4.6)	32 (5)	10	4 conditions 1. Uninterrupted sitting a. Duration: 9 hours 2. Physical activity bouts a. Type: WALK b. Duration: 30 minutes c. Frequency: single bout d. Intensity: 3.3 mph, 65–75% HR_max_ (moderate) 3. Physical activity bouts a. Type: WALK b. Duration: 2 minutes c. Frequency: every 20 minutes d. Intensity: 3 mph, 53% ± 5 HR_max_ (moderate) 4. Physical activity bouts a. Type: WALK b. Duration: 2 minutes c. Frequency: every hour d. Intensity: average 79% HR_max_ (vigorous)	I. Hypertension II. Dysglycemia	**Systolic blood pressure:** Baseline SBP did not differ between conditions. 30-min MOD significantly reduced 18.7 hour SBP (*p* < 0.05) compared to sitting **Mean arterial pressure:** 30-min MOD significantly reduced 18.7 hour MAP (*p* < 0.05) compared to sitting **Postprandial glucose:** All 3 conditions (30-min MOD, 2-min MOD, 2-min VIG) significantly reduced 18.7 hour glucose compared to uninterrupted sitting (all *p* < 0.01). 30-min MOD was significantly lower compared to 2-min VIG and 2-min MOD (all *p* < 0.01) and 2-min MOD was significantly lower than 2-min VIG (*p* < 0.01)
Barone Gibbs et al. (2017) *No trial registry number*	Randomised crossover trial	Overweight/obese adults with pre to stage 1 hypertension 9 females, 16 males BMI = 31.9 (5)	42 (12)	25	2 conditions 1. Uninterrupted sitting a. Duration: 9 hour 2. Physical activity bouts a. Type: STAND b. Duration: 30 minutes c. Frequency: every 30 minutes d. Intensity: not specified	I. Hypertension	**Systolic blood pressure:** No significant differences **Diastolic blood pressure:** STAND significantly reduced DBP compared with uninterrupted sitting (*p* = 0.02) **Mean arterial pressure:** STAND significantly reduced MAP compared to uninterrupted sitting (*p* = 0.03)
Hawari et al. (2016) *No trial registry number*	Randomised crossover trial	Overweight/obese, normoglycaemic males BMI = 28.3 (2.8)	33 (13)	10	3 conditions 1. Uninterrupted sitting a. Duration: 8 hours 2. Physical activity bouts a. Type: STAND b. Duration: 15 minutes c. Frequency: every 15 minutes d. Intensity: not specified 3. Physical activity bouts a. Type: STAND b. Duration: 1.5 minutes c. Frequency: every 30 minutes d. Intensity: not specified	I. Dysglycemia	**Postprandial glucose:** No significant differences
McCarthy et al. (2017) *NCT02909894*	Randomised crossover trial	Obese and inactive adults at risk of type 2 diabetes 7 females, 6 males BMI = 33.8 (3.8)	66 (6)	13	2 conditions 1. Uninterrupted sitting a. Duration: 7.5 hours 2. Physical activity bouts a. Type: ARM ERGOMETRY b. Duration: 5 minutes c. Frequency: every 30 minutes d. Intensity: 3km.h^-1^ (light)	II. Dysglycemia	**Postprandial glucose:** Glucose iAUC was significantly lower during the arm ergometer bouts compared to sitting (*p* < 0.01)
Holmstrup et al. (2014) *No trial registry number*	Randomised crossover trial	Obese, young adults with impaired fasting glucose 3 females, 8 males BMI = 34.0 (SD not reported)	25 (SD not reported)	11	3 conditions 1. Uninterrupted sitting a. Duration: 12 hours 2. Physical activity bouts a. Type: WALK b. Duration: 60 minutes c. Frequency: single bout d. Intensity: 60–65% VO2peak 3. Physical activity bouts a. Type: WALK b. Duration: 5 minutes c. Frequency: every hour d. Intensity: 60–65% VO2peak	II. Dysglycemia	**Postprandial glucose:** Glucose iAUC (12 hour) significantly different between conditions (*p* = 0.021) with a higher glucose in the EX condition (SIT *vs*. EX, *p* = 0.04; EX *vs*. EX-INT, *p* = 0.05)
**Type 2 diabetes**							
Dempsey et al. (2016) *ACTRN12613000576729* Dempsey et al. (2016) Dempsey et al. (2017)	Randomised crossover trial	Type 2 diabetic, overweight/obese and inactive adults 10 females, 14 males BMI = 33 (3.4)	62 (6)	24	3 conditions 1. Uninterrupted sitting a. Duration: 7 hours 2. Physical activity bouts a. Type: WALK b. Duration: 3 minutes c. Frequency: every 30 minutes d. Intensity: 3.2 km.h^-1^ (light) 3. Physical activity bouts a. Type: Simple resistance activities (SRA; body weight half squats, calf raises, gluteal contractions and knee raises) b. Duration: 3 minutes c. Frequency: every 30 min d. Intensity: not specified	II. Dsyglycemia	**Postprandial glucose:** Net 7 hours glucose iAUC was significantly reduced following both WALK and SRA (*p* < 0.01) compared to sitting. WALK bouts significantly reduced iAUC in both sexes compared to sitting, with a greater reduction seen in women (*p* = 0.05)
						I. Hypertension	**Systolic blood pressure:** Resting SBP was significantly reduced following both WALK and SRA (*p* < 0.01), with SRA having a greater effect compared to WALK (*p* < 0.05) **Diastolic blood pressure:** Resting DBP was significantly reduced following both WALK and SRA (*p* < 0.01), with SRA having a greater effect compared to WALK (*p* < 0.05)
						II. Dsyglycemia	**Postprandial glucose:** *Over 22 hours*—WALK and SRA significantly lowered mean glucose, time spent in hyperglycaemia and tAUC compared to uninterrupted sitting (all *p* < 0.01) *Glycaemic control (postprandial)—*WALK and SRA significantly reduced mean glucose, time in hyperglycaemia and iAUC in comparison to uninterrupted sitting (*p* < 0.05) *Nocturnal glycaemic control*—tAUC, mean glucose and time hyperglycaemia were significantly lower during sleep, following SRA and WALK (*p* < 0.01), with mean glucose remaining significantly reduced the morning after (*p* < 0.01)
Dijk et al. (2013) *NCT00945165*	Randomised crossover trial	Type 2 diabetic males BMI = 29.5 (0.9)	64 (1)	20	3 conditions 1. Uninterrupted sitting a. Duration: 12 hours 2. Physical activity bouts a. Type: ADL (light strolling) b. Duration: 15 minutes c. Frequency: after each meal (09:15, 13:15, 17:45) d. Intensity: ~3 METs 3. Physical activity bouts a. Type: CYCLE b. Duration: 45 minutes c. Frequency: single bout d. Intensity: 50% maximal workload capacity, ~6 METs (moderate)	II. Dysglycemia	**Postprandial glucose:** *24 hour glycaemic control*—A 45 min bout of cycling significantly reduced 24 hours glucose and the incidence of hyperglycaemia compared to uninterrupted sitting (both *p* < 0.01). *Postprandial glycaemic control*–The single cycling bout significantly reduced the cumulative glucose (*p* < 0.01) and glycaemic response (*p* < 0.05) to all meals compared to uninterrupted sitting. ADL also significantly reduced the cumulative glucose response to all meals compared to uninterrupted sitting (*p* < 0.05)
**Postmenopausal**							
Kerr et al. (2017) *NCT02743286*	Randomised crossover trial	Postmenopausal, overweight/obese and sedentary women BMI = 30.6 (4.2)	66 (9)	10	4 conditions 1. Uninterrupted sitting a. Duration: 5 hours 2. Physical activity bouts a. Type: STAND b. Duration: 2 minutes c. Frequency: every 20 minutes d. Intensity: not specified 3. Physical activity bouts a. Type: WALK b. Duration: 2 minutes c. Frequency: every hour d. Intensity: light 4. Physical activity bouts a. Type: STAND b. Duration: 10 minutes c. Frequency: every hour d. Intensity: not specified	I. Hypertension II. Dysglycemia	**Systolic blood pressure:** No significant differences **Diastolic blood pressure:** No significant differences **Postprandial glucose:** No significant differences in iAUC
Miyashita et al. (2016) *No trial registry number*	Randomised crossover trial	Postmenopausal women BMI = 24 (2.9)	69 (3)	15	3 conditions 1. Uninterrupted sitting a. Duration: 8 hours 2. Physical activity bouts a. Type: WALK b. Duration: 1.5 minutes c. Frequency: every 15 minutes d. Intensity: average 3.7 ± 11 km.h^-1^ 3. Physical activity bouts a. Type: WALK b. Duration: 30 minutes c. Frequency: Single bout d. Intensity: average 3.7 ± 11 km.h^-1^	II. Dysglycemia	**Postprandial glucose:** No significant differences compared to uninterrupted sitting. tAUC (*p* < 0.01) and iAUC (*p* = 0.01) were greater during the continuous walk compared to the regular walk
Henson et al. (2016) *NCT02135172*	Randomised crossover study	Postmenopausal, overweight/obese dysglycemic women BMI = 32.9 (4.7)	67 (5)	22	3 conditions each conducted over 2 days (Day 1; activity bout condition, Day 2; uninterrupted sitting) 1. Uninterrupted sitting a. Duration: 7.5 hours 2. Physical activity bouts a. Type: STAND b. Duration: 5 minutes c. Frequency: every 30 minutes d. Intensity: stand in a fixed position 3. Physical activity bouts a. Type: WALK b. Duration: 5 minutes c. Frequency: every 30 minutes d. Intensity: 3 km.h-1, RPE of 10 (light)	II. Dysglycemia	**Postprandial glucose:** *Day 1* –Standing and walking bouts significantly reduced glucose iAUC compared to uninterrupted sitting (*p* = 0.02 and *p* = 0.01, respectively) *Day 2* –Standing and walking bouts completed on Day 1 significantly reduced glucose iAUC on Day 2 compared to uninterrupted sitting (*p* = 0.04 and *p* = 0.03, respectively)
**Stroke**							
English et al. (2018) *ANZTR12615001189516* English et al. (2018)	Randomised crossover trial	Stroke survivors > 3 months and < 10 years post stroke 9 females, 10 males BMI = 29.9 (5.1)	68 (10)	19	3 conditions 1. Uninterrupted sitting a. Duration: 8 hours 2. Physical activity bouts a. Type: Standing exercises (STAND-EX; marching on spot, small amplitude squats, calf-raises) b. Duration: 3 minutes c. Frequency: every 30 minutes c. Intensity: RPE—2 (1), HR-73 (10) (light) 3. Physical activity bouts a. Type: WALK b. Duration: 3 minutes c. Frequency: every 30 minutes d. Intensity: RPE—1 (1), HR -73 (11.3) (light)	I. Hypertension	**Systolic blood pressure:** STAND-EX significantly reduced SBP compared with SIT **Diastolic blood pressure:** No significant differences (*p* = 0.45)
						II. Dysglycemia	**Postprandial glucose:** No siginificant effect of experimental condition on glucose (*p* = 0.56)

ADL, activities of daily living; AUC, area under the curve; BMI, body mass index; DBP, diastolic blood pressure; HDL, high density lipoprotein; HR, heart rate; HR_max_, maximum heart rate; HRR, heart rate reserve; iAUC, incremental area under the curve; LDL, low density lipoprotein; MAP, mean arterial pressure; MET, metabolic equivalent; RPE, rating of perceived exertion (Borg); SBP, systolic blood pressure; SEM, standard error of the mean; tAUC, total area under the curve; VO2_max_, maximal oxygen uptake; VO2_peak_, peak oxygen uptake. All data is represented as mean (standard deviation) unless otherwise stated

*Data reported as mean (range; low to high)

Ten trials (12 articles [12, 14, 15, 33, 35, 37, 38, 42, 49, 50, 54, 55]) specifically recruited overweight/obese adults (Table 3). Seven trials (9 articles [12, 15, 33, 35, 37, 38, 42, 50, 55]) included obese adults (BMI of ≥ 30 kg.m^-2^ and < 34.9 kg.m^-2^) and three trials [14, 49, 54] included overweight adults. The age ranges of participants in these trials varied and included young adults (four trials [14, 33, 54, 55]), middle aged adults (> 35 and < 65 years) (five trials, [12, 15, 35, 37, 42, 49, 50]) and older adults (≥ 65 years) (one trial [38]).

**Table 3:** Outcomes associated with first and recurrent stroke risk factors (N = number of trials measuring this risk factor).

Author (year) Trial ID	Healthy (N = trials)	Overweight/obese (N = trials)	Type 2 diabetic (N = trials)	Postmenopausal (N = trials)	Stroke (N = trials)	Total number of trials
**Hypertension** Systolic blood pressure Diastolic blood pressure Mean arterial pressure	N = 2 1 1 1	N = 5 5 4 3	N = 1 1 1 -	N = 1 1 1 -	N = 1 1 1 -	N = 10 9 8 4
**Dysglycemia** Fasting glucose Postprandial glucose Impaired glucose tolerance HBA1c or diabetes	N = 12 3 12 - -	N = 8 2 8 - -	N = 2 - 2 - -	N = 3 - 3 - -	N = 1 - 1 - -	N = 26 5 26 - -
**Anthropometric** BMI Waist-to-hip ratio Weight	N = 0 - - -	N = 1 - - 1	N = 0 - - -	N = 0 - - -	N = 0 - - -	N = 1 - - 1
**Hypercholesterolemia** Total cholesterol HDL cholesterol LDL cholesterol Apo B/Apo A 1	N = 4 4 4 3 -	N = 0 - - - -	N = 0 - - - -	N = 0 - - - -	N = 0 - - - -	N = 4 4 4 3 -
**Behavioural** Diet Smoking Low physical activity Alcohol	N = 0 - - - -	N = 0 - - - -	N = 0 - - - -	N = 0 - - - -	N = 0 - - - -	N = 0 - - - -
**Psychosocial** Stress Depression	N = 0 - -	N = 0 - -	N = 0 - -	N = 0 - -	N = 0 - -	N = 0 - -
**Cardiac** Atrial fibrillation Myocardial infarction	N = 0 - -	N = 0 - -	N = 0 - -	N = 0 - -	N = 0 - -	N = 0 - -

ApoB/ApoA 1 ratio, apolipoproteinB/apolipoproteinA1 ratio; BMI, body mass index; HbA1c, glycated haemoglobin; HDL-C, high density lipoprotein cholesterol; LDL-C, low density lipoprotein cholesterol

Only two trials (four articles) recruited individuals with type 2 diabetes [16, 44–46] (Table 2), where one included overweight adults with type 2 diabetes [46] and the other included obese adults with type 2 diabetes [16, 44, 45]. All trials were completed in middle age adults with a range of 62 ± 6 years to 64 ± 1 years.

Three trials [18, 34, 58] recruited postmenopausal women (aged > 65 years) (Table 2) who were normal weight [58] or overweight [18, 34]. Only one trial presented data for stroke survivors who were 0.25 to 10 years post-stroke, older adults (68 ± 2 years) and overweight [20, 30].

### Characteristics of the physical activity bouts

The type, duration, frequency and intensity of activity bouts varied across trials, as did the length of the intervention periods (see Table 2). Assessments were completed either on a single day or over multiple days.

### Stroke and recurrent stroke risk factors measured

Ten trials included measures of hypertension [14–16, 20, 30, 33, 34, 41, 42, 50, 52], 26 included measures of dysglycemia [12, 18–20, 30, 32–35, 37–41, 43–51, 53–58], one included measures of anthropometric risk factors [49], and four trials included measures of hypercholesterolemia [31, 32, 41, 43]. No trials presented behavioural, psychosocial or cardiac risk factors (Table 3).

### Effects of physical activity bouts on stroke or recurrent stroke risk factors

#### Healthy participants

Outcomes associated with hypertension (mean arterial pressure [52], systolic blood pressure and diastolic blood pressure [41]) were examined in two trials involving healthy adults. Regular short bouts of standing, walking or calisthenics did not significantly change mean arterial pressure, systolic blood pressure or diastolic blood pressure when compared to uninterrupted sitting.

Nine trials measured dysglycemia over a single day [19, 32, 39–41, 43, 48, 51, 53]. Three trials found no significant effects of physical activity bouts on postprandial glucose compared to uninterrupted sitting [32, 40, 53]. The remaining five trials [19, 39, 41, 48, 51] observed significant reductions in postprandial glucose with varying physical activity bout types (walking and standing), durations (1 minute 40 seconds to 5 minutes) and frequencies (every 20 to 30 minutes). Another trial, taking place over 27 hours, found sitting interrupted by regular standing bouts reduced postprandial glucose on the day of and the morning after the intervention [43].

Three trials measured dysglycemia over multiple days (two [47, 57] to four [56] days) using measures of fasting and/or postprandial plasma glucose. Fasting glucose was measured in all three trials and showed no significant between condition differences for physical activity bouts compared to prolonged sitting. With regards to postprandial glucose, one trial found no significant between condition differences [47], while two trials found significant reductions in postprandial glucose following a single bout of walking (30 minute) [57] and running (60 minute) [56] (completed the day before glucose assessments). Kim et al. [56] also found a significant reduction in postprandial glucose the day after intermittent bouts of walking.

Four trials measured outcomes associated with hypercholesterolemia and found no significant between condition differences in total cholesterol, low density lipoprotein (LDL) cholesterol [31] and high density lipoprotein (HDL) cholesterol [32, 41, 43], expect for one trial which found a reduction in HDL cholesterol [31]. Engeroff et al. [31] found frequent bouts of cycling (6 minutes every 40 minutes) had a negative impact on HDL cholesterol.

#### Overweight/obese participants

A total of five trials [14, 15, 33, 42, 50] measured outcomes associated with hypertension in overweight/obese participants. Systolic blood pressure was measured in all five trials, while diastolic blood pressure was measured in four trials [14, 15, 42, 50] and mean arterial pressure in three trials [15, 33, 42]. In two of the five trials, systolic blood pressure did not significantly differ between conditions [42, 50]. The remaining three trials found significant reductions in systolic blood pressure following frequent bouts (2 to 30 minutes every 20 to 60 minutes) of physical activity (light to moderate intensity walking, standing and cycling) [14, 15] and a single bout of moderate-intensity walking (30 minutes) [33]. Diastolic blood pressure did not significantly reduce in one trial [50], but was reduced in the remaining three trials following different types (walking, cycling and standing), durations (2 minutes to 30 minutes), frequencies (every 20 to 60 minutes) and intensities (light to moderate) of physical activity bouts [14, 15, 42]. In two trials, mean arterial pressure was significantly reduced following frequent standing bouts (30 minutes every 30 minutes) [42] and a single bout of moderate-intensity walking (30 minutes) [33]. One trial found no significant condition differences compared to sitting for mean arterial pressure [15].

Dysglycemia over a single day (postprandial glucose) was measured in six trials [12, 33, 37, 38, 50, 54, 55]. Three trials found no significant reductions in postprandial glucose following activity bouts compared to prolonged sitting [50, 54, 55]. However, the trial by Homlstrup et al. [55] found a significant increase in postprandial glucose iAUC following a single bout of walking compared to uninterrupted sitting. The remaining three trials [12, 33, 37, 38] found significant between condition improvements in postprandial glucose with varying physical activity bout types (arm ergometry, walking, standing), durations (2 to 30 minutes), frequencies (20 to 60 minutes) and intensities (light, moderate and vigorous).

Dysglycemia (fasting and postprandial glucose) was also measured over multiple (three to five) days. Two trials found regular bouts of standing (30 minutes every 30 minutes) [49] and light-intensity walking (2 minutes every 20 minutes) [35] significantly reduced postprandial glucose compared to prolonged sitting. No significant differences were found for fasting glucose responses in both trials.

The only trial to assess anthropometric risk factors [49] found no significant effect of conditions on weight loss over a five day period.

#### Type 2 diabetes participants

In the only trial investigating outcome measures related to hypertension in participants with type 2 diabetes [16], bouts of light-intensity walking and simple resistance activities (3 minutes every 30 minutes) significantly lowered systolic blood pressure and diastolic blood pressure response in comparison to prolonged sitting.

Postprandial glucose was the only marker associated with dsyglycemia to be measured in this population, and was investigated in two trials [44–46]. One trial found that interrupting prolonged sitting with frequent bouts of activities of daily living (15 minutes completed after meal) and a 45 minute single bout of cycling, lowered postprandial glucose response compared to sitting [46]. The other trial by Dempsey et al. [44, 45] found a significant reduction in postprandial glucose following 3 minutes of walking and simple resistance activities every 20 minutes, when compared to uninterrupted sitting.

#### Postmenopausal participants

One trial found no significant difference between conditions for systolic and diastolic blood pressure in postmenopausal women [34].

Postprandial glucose was measured in a total of three trials [18, 34, 58]. Two trials found no significant effect of physical activity bouts compared to prolonged sitting on postprandial glucose over one day [34, 58]. One trial, completed over two days, found postprandial glucose to be significantly reduced on both days following 5 minutes of standing and walking every 30 minutes completed on Day 1 [18].

#### Stroke participants

The only trial in stroke saw a significant reduction in systolic blood pressure following 3 minutes of standing activity, every 30 minutes, in comparison to uninterrupted sitting [20].

Frequent activity bouts (standing or walking) did not significantly alter postprandial glucose compared to uninterrupted sitting [30].

## Discussion

This review has synthesised available evidence regarding the effect of interrupting prolonged sitting with frequent bouts of physical activity or standing on risk factors for first or recurrent stroke. A total of 15 trials recruited participants at risk of first stroke (overweight/obese, type 2 diabetes, postmenopausal women/older adults) and one trial in participants at risk of recurrent stroke (one trial; stroke survivors). Four key first or recurrent stroke risk factors (hypertension, hypercholesterolaemia, dysglycemia and weight loss) were measured. In populations identified at high risk of first or recurrent stroke, interrupting prolonged sitting with frequent bouts of physical activity or standing tended to show beneficial effects on outcomes associated with hypertension and dysglycemia, but not on hypercholesterolaemia and weight loss.

With regard to the relevance to stroke risk, a large proportion of trials were conducted in participants characterised as being at risk of first stroke. In the majority of these trials, participants were characterised as overweight (13 trials) or obese (4 trials). This is highly relevant given that an elevated BMI is recognised as a prominent risk factor for stroke [27]. The incidence of a first stroke is also greater with advancing age [59, 60] and in individuals with type 2 diabetes [29]. However, only four trials included participants characterised as older adults (3 trials in postmenopausal women and one trial in overweight/obese older adults) and only two trials included participants with type 2 diabetes, representing a limited number of trials in these high risk population groups. Furthermore, no trials focused on older adults in the healthy population group. Overall, the characteristics of participants in the 17 trials considered at risk of stroke were representative of the participant characteristics in the trial by English et al. [20, 30].

### Outcomes associated with hypertension

Hypertension is the foremost risk factor associated with first and recurrent stroke, with elevated systolic blood pressure recognised as the primary measure of hypertension [8, 9, 27]. Interrupting prolonged sitting with frequent bouts of physical activity improved systolic blood pressure in the majority of participants at risk of a stroke (overweight/obese or those who have type 2 diabetes), following predominately short bouts of light- to moderate-intensity physical activity (walking, cycling, standing and simple body weight exercises) [14–16, 33]. More importantly, in the one trial completed in stroke survivors, systolic blood pressure was significantly reduced in response to frequent bouts of light-intensity exercise while standing [20]. The light- to moderate-intensity of physical activity bouts prescribed are comparable to the recommendations from the American Heart Association and American Stroke Association for promoting physical activity after stroke [61]. Additional measures of hypertension, such as diastolic blood pressure and mean arterial pressure [28, 29], were also positively influenced following frequent bouts of physical activity [14–16, 33, 42]. The overall improvements in blood pressure response in these high risk populations are encouraging, even allowing for the small number of trials measuring hypertension outcomes. Dempsey et al. [62] confirms the potential benefits of interrupting prolonged sitting in controlling blood pressure in population groups at risk. To build on the promising findings in this review, further work is needed to develop and test clinically meaningful interventions of frequent bouts of physical activity or standing to reduce outcomes of hypertension in populations at greater risk of first and recurrent stroke. The assessment of ambulatory blood pressure in future work would also add to the clinical importance of results.

### Outcomes of dysglycemia

Outcomes of dysglycemia are similarly important risk factors related to first and recurrent stroke [8, 9]. Two recent reviews investigating risk factors for first stroke assessed dysglycemia by measuring either fasting plasma glucose or HbA1c [8, 27]. With a focus on fasting plasma glucose, frequent bouts of physical activity appeared to be ineffective at reducing fasting plasma glucose in participants at risk of stroke (overweight/obese) [35, 49]. This response is consistent with previous literature investigating the short-term effect of exercise on fasting glucose control [63, 64]. However, fasting plasma glucose does not provide an indication of the fluctuations in glucose concentrations over a day. Instead, postprandial glucose is used as an indicator for glycaemic control in the hours after a meal and is an associated risk factor for first and recurrent stroke risk [8, 29, 65]. Frequent bouts of physical activity or standing led to significant reductions in postprandial glucose response in the majority of trials involving overweight/obese participants and in those with type 2 diabetes [12, 33, 35, 37, 38, 44–46, 49]. In trials of postmenopausal women, only one of three trials found a significant improvement in postprandial glucose following frequent bouts of standing and walking [18]. The improvement found in the trial by Henson et al. [18] could be due to participants being dysglycemic. Likewise, in the community dwelling stroke survivors in the study by English et al. [30], postprandial glucose was not affected by frequent bouts of physical activity. Nevertheless, future trials are needed to further understand the effects of frequent bouts of physical activity on the postprandial glucose response in stroke survivors. Additional trials investigating fasting plasma glucose are further required in populations at risk of first and recurrent stroke.

### Outcomes of anthropometric measures

The risk of first and recurrent stroke is also elevated in individuals with a high BMI [9, 27]. Only one trial involving overweight/obese participants investigated the effects of frequent bouts of standing on anthropometric risk factors (weight management) [49], with no beneficial improvements found. The absence of a response could be in part due to the short duration of the trial (5 days), in conjunction with an insufficient increase in energy expenditure to produce a sufficient energy deficit for weight loss [66]. Trials of greater duration and intensity would be required to explore the effects of frequent bouts of physical activity upon weight management.

### Outcomes of hypercholesterolaemia

Hypercholesterolaemia accounts for a small proportion of the stroke risk (5%) as estimated in the global burden of disease trial [27]. With regards to recurrent stroke risk, LDL and HDL cholesterol are important risk factors, with LDL cholesterol recognised as a more prominent risk factor [9]. However, outcomes of hypercholesterolaemia were only measured in the healthy population (four trials) [31, 32, 41, 43]. Although no significant beneficial effects of frequent bouts of physical activity or standing were found, the trial by Engeroff et al. [31] found significant trial x time interactions. The pre- to post-intervention changes in total cholesterol were significantly different between frequent bouts of physical activity (negative change) and a single bout of physical activity (positive change), but not uninterrupted sitting (positive change). HDL cholesterol during the frequent bouts of physical activity differed significantly (negative change) compared to a single bout of physical activity (positive change) and uninterrupted sitting (positive change). Reduced HDL cholesterol concentrations are linked to an increased risk of having a stroke [8, 67], although conversely, O’Donnell et al. [8] found a direct relationship between elevated HDL cholesterol and risk of an intracerebral haemorrhagic stroke. However, the trial by Engeroff et al. [31] was conducted in young, normal weight adults who are at a reduced risk of having a stroke. Given the importance of hypercholesterolaemia on stroke risk, more trials are required in populations at high risk of stroke to identify the effects of frequent bouts of physical activity on outcomes of hypercholesterolaemia.

Strengths of this review are that it focused on outcome measures associated with first and recurrent stroke risk. Our definition of supervised interventions may be considered a limitation of this review. A small proportion of trials did not use the terms ‘supervised’, ‘monitored’ or ‘observed’ and simply stated that trials were completed in a laboratory or research facility. However, protocol adherence was monitored in the majority of trials with activity monitors, giving an indication that bouts of physical activity were adhered to in the trials. Additionally, this review was designed to provide a broad overview of experimental trials on breaking up prolonged sitting time with frequent bouts of physical activity or standing and therefore did not report on the magnitude of effect of interventions. While we are confident that we identified all relevant literature at the time of searching, this is a rapidly expanding field and further papers may have been published since.

In conclusion, there is consistent evidence from a number of trials that breaking up prolonged sitting with frequent bouts of physical activity or standing has positive effects on some stroke risk factors (hypertension and dysglycemia) in population groups at risk of a stroke. In the only study of people with stroke, positive effects were seen for hypertension only. Given hypertension is the leading risk factor for stroke, this review provides a solid rationale for further work to determine the optimal frequency, intensity and duration of physical activity bouts to reduce blood pressure, and if effects are maintained.

## Supporting information

S1 Appendix MEDLINE search strategy. (DOCX)

S2 Appendix PRISMA checklist. (DOC)
