# Supplementary material for: What is the effect of interrupting prolonged sitting with frequent bouts of physical activity or standing on first or recurrent stroke risk factors? A scoping review
Source: PLoS One. 2019 Jun 13;14(6):e0217981. doi: 10.1371/journal.pone.0217981 (PMC6563984; doi:10.1371/journal.pone.0217981)
Supplement: S1 Appendix — (DOCX) [file pone.0217981.s001.docx]

**S1 Appendix – MEDLINE Search Strategy:**

--------------------------------------------------------------------------------

1 Sedentary Lifestyle/

2 sedentary behavio?r*.tw.

3 (sedentary adj3 (activit* or behavio?r* or state or time)).tw.

4 ((sit* or sat or lay or lying or sedentary or reclin* or stationary*) adj3 (time or period* or bout* or duration* or prolong* or pattern* or uninterrupt* or continuous)).tw.

5 Exercise/

6 (break* in sedentary behavio?r* or BSB).tw.

7 (break* adj3 (up or sedentary or prolong* or sit* or behavio?r*)).tw.

8 (interrupt* adj2 (prolong* or sedentary or sit*)).tw.

9 stationary behavio?r*.tw.

10 activity behavio?r*.tw.

11 (walk* adj2 (activit* or bout*)).tw.

12 (stand* adj2 (activit* or bout*)).tw.

13 5 or 6 or 7 or 8 or 10 or 11 or 12

14 Posture/ph [Physiology]

15 1 or 2 or 3 or 4 or 9 or 14

16 13 and 15

17 limit 16 to (english language and humans)
